# Performance of Cone Beam Computed Tomography Systems in Visualizing the Cortical Plate in 3D Image Reconstruction: An *In Vitro* Study

**DOI:** 10.2174/1874210601812010586

**Published:** 2018-08-31

**Authors:** Abbas Shokri, Mohammad Reza Jamalpour, Amir Eskandarloo, Mostafa Godiny, Payam Amini, Atefeh Khavid

**Affiliations:** 1 Department of Oral and Maxillofacial Radiology, Dental Research Center, Dental School, Hamadan University of Medical Sciences, Hamadan, Iran.; 2 Department of Oral and Maxillofacial Surgery, Dental Implant Research Center, Dental School, Hamadan University of Medical Sciences, Hamadan, Iran.; 3 Department of Oral and Maxillofacial Radiology, Dental School, Hamadan University of Medical Sciences, Hamadan, Iran.; 4 Department of Endodontics, Dental school , Kermanshah University of Medical Sciences, Kermanshah, Iran.; 5 Department of Epidemiology and Reproductive Health, Reproductive Epidemiology Research Center, Royan Institute for Reproductive Biomedicine, ACECR, Tehran, Iran.; 6 Department of Oral and Maxillofacial Radiology, Dental School, Kermanshah University of Medical Sciences, Kermanshah, Iran.

**Keywords:** Cone-Beam Computed Tomography (CBCT), Cortical bone, Imaging, Three-dimensional, Image processing, Computer-assisted

## Abstract

**Introduction:**

Cortical bone is an important anatomical structure and its thickness needs to be determined prior to many dental procedures to ensure treatment success. Imaging modalities are necessarily used in dentistry for treatment planning and dental procedures. Three-dimensional image reconstruction not only provides visual information but also enables accurate measurement of anatomical structures; thus, it is necessarily required for maxillofacial examination and in case of skeletal problems in this region.

**Aims:**

This study aimed to assess the ability of three Cone Beam Computed Tomography (CBCT) systems including Cranex 3D, NewTom 3G and 3D Promax for Three-Dimensional (3D) image reconstruction of the cortical plate with variable thicknesses.

**Methods:**

Depending on the cortical bone thickness, samples were evaluated in three groups of ≤ 0. 5 mm, 0.6 -1 mm and 1.1-1.5 mm cortical bone thickness. The CBCT scans were obtained from each sample using three systems, their respective FOVs, and 3D scans were reconstructed using their software programs. Two observers viewed the images twice with a two-week interval. The ability of each system in the 3D reconstruction of different thicknesses of cortical bone was determined based on its visualization on the scans. The data were analyzed using *SPSS* and Kappa test.

**Results:**

The three systems showed the greatest difference in the 3D reconstruction of cortical bone with < 0.5 mm thickness. Cranex 3D with 4×6 cm^2^ FOV had the highest and 3D Promax with 8×8 cm^2^ FOV had the lowest efficacy for 3D reconstruction of cortical bone. Cranex 3D with 4×6 cm^2^ and 6×8 cm^2^ FOVs and NewTom 3G with 5×5 cm^2^ and 8×5 cm^2^ FOVs showed significantly higher efficacy for 3D reconstruction of cortical bone with 0.6-1mm thickness while 3D Promax followed by NewTom 3G with 8×8 cm^2^ FOV had the lowest efficacy for this purpose.

**Conclusion:**

Most CBCT systems have high efficacy for 3D image reconstruction of cortical bone with thicknesses over 1 mm while they have poor efficacy for image reconstruction of cortical bone with less than 0.5 mm thickness. Thus, for accurate visualization of anatomical structures on CBCT scans, systems with smaller FOVs and consequently smaller voxel size are preferred.

## 1 INTRODUCTION

Imaging modalities are necessarily used in dentistry for treatment planning and dental procedures. Advances in science facilitate the use of modern technologies. Advanced imaging techniques such as Computed Tomography (CT) revolutionized medicine and dentistry; however, the application of CT is limited in dentistry due to high cost, large size of equipment and high patient radiation dose [1].

Cone Beam Computed Tomography (CBCT) was introduced as a standard alternative to CT for diagnostic and therapeutic purposes. It is extensively used for 3D image reconstruction of the maxillofacial region and plays an important role in dental diagnosis and treatment planning [2]. Three-dimensional image reconstruction not only provides visual information but also enables accurate measurement of anatomical structures; thus, it is necessarily required for maxillofacial examination and in case of skeletal problems in this region [3].

Cortical bone is an important anatomical structure and its thickness needs to be determined prior to many dental procedures to ensure treatment success. Primary stability of implants, which is critical for adequate osseointegration, depends on the thickness of cortical bone adjacent to the implant. Thus, it is imperative to find regions of jawbone with an adequate thickness of cortical bone for implant placement. In orthodontics, use of mini-implants to provide orthodontic anchorage is a relatively new treatment modality. Determining a proper location for insertion of mini-implants also depends on the presence of adequate thickness of cortical bone in the area because, in case of the insufficient thickness of cortical bone at the site, fenestration may occur during orthodontic treatment and following load application [4].

Conventional Two-Dimensional (2D) radiographs cannot accurately determine the thickness and volume of cortical bone for placement of mini-implants. The inadequate thickness of cortical bone at the site significantly increases the risk of premature loosening of mini-implants [1, 4].

Moreover, cortical bone needs to be radiographically examined prior to orthodontic treatment to assess the possible presence of impacted teeth. If the orthodontic treatment plan includes forced eruption of impacted teeth, 3D image reconstruction by CBCT can significantly enhance the visualization of cortical plate at the site and help to predict the success of treatment. Not paying attention to the absence of cortical bone or its insufficient thickness at the site of treatment may eventually result in the hopeless prognosis of tooth following the application of orthodontic forces [4, 5].

Oral and maxillofacial surgeons most commonly benefit from the algorithms of 3D image reconstruction. In some maxillofacial surgical procedures especially those associated with the use of grafts and particularly ramus bone grafts, selection of surgical site highly depends on the presence of adequate thickness of cortical bone and adequate quality of bone in the area because normally ramus bone grafts have a long length and very thin thickness and it is important to ensure adequate thickness of cortical bone at the graft recipient site preoperatively [6].

One common problem encountered by maxillofacial surgeons and radiologists when assessing anatomical structures and pathological conditions of the maxillofacial region on conventional radiographs is that the cortical plate has not been well visualized in the reconstructed images, which leads to misdiagnosis of inadequate thickness of cortical bone or bone perforation.; thus, accurate radiographic visualization of cortical bone can significantly affect treatment planning [7].

The CBCT scans are necessarily required for more accurate visualization of jawbones in many patients. Recent advances in CBCT systems have been noticeable. It appears that different CBCT systems have variable efficacy for 3D image reconstruction [7].

The quality of 3D image reconstruction by these systems and their efficacy in visualization of maxillofacial structures are affected by several factors including the type of system used for data acquisition, Field Of View (FOV), selection of scanning parameters and 3D reconstruction algorithms of each system [8].

The accuracy of 3D image reconstruction by CBCT has not been well investigated in the literature (this citation is not accurate) [9].

Considering the importance of cortical bone visualization on 3D CBCT scans and the variable ability of CBCT systems in terms of 3D image reconstruction, it is important to find the system with the best efficacy for visualization of cortical bone, especially in thin thicknesses. Moreover, it is imperative to know the minimum thickness of cortical bone visualized by the software programs of different CBCT systems and detectable on the reconstructed 3D images.

Concerning the significance of the aforementioned topics and since no study has evaluated the ability of CBCT systems for 3D image reconstruction of different thicknesses of cortical bone, this study sought to assess the efficacy of three CBCT systems for 3D image reconstruction of variable thicknesses of cortical bone.

## 2 MATERIALS AND METHODS

This study was performed in accordance with the Declaration of Helsinki and was approved by the ethics committee of Hamadan University of Medical Sciences (ECHUMS) issued in 2016 (Grant number: EC-16-35-9-220).

Rib bone of a freshly slaughtered cow was used, comprising of a cortical outer layer and a cancellous core; it enabled cutting very thin slices of cortical bone. First, the bones were sectioned using a saw (Lamico, Tehran, Iran) and a cutting disc (Lamico, Tehran, Iran). Next, a milling machine (Lamico, Tehran, Iran) was used to cut bone sections into cubes with 2×2 cm^2^ dimensions. Samples were cut in such a way that a layer of cortical bone was present in their buccal, lingual and superior surface and they contained cancellous bone at the center. The bones were kept in a freezer during the study period to remain hydrated. According to the required cortical bone thickness, the samples were prepared in three groups (n=24):

Group 1. Samples with a ≤ 0.5 mm thickness of cortical bone
Group 2. Samples with a 0.6-1 mm thickness of cortical bone
Group 3. Samples with 1.1 to 1.5 mm thickness of cortical bone

All samples were coded and each one was placed independently in the CBCT systems and scanned. Cross-sectional views of cortical bone thickness in the buccal surface were obtained using the CBCT system software. In cases where the thickness of cortical bone was greater than the required thickness in groups, the thickness was decreased by a milling machine and CBCT scans were obtained to ensure the desired thickness of cortical bone in samples.

For soft tissue simulation, a model of the mandible was used and wax layers were shaped in the form of the mandible [1, 9]. Inside each model, six samples were randomly mounted and fixed. A total of 12 simulated mandibular models were fabricated as such.

### 2.1 CBCT Examination

All mandibular models with samples mounted in them were scanned using the three CBCT systems. In other words, each sample was scanned with the three CBCT systems with their respective FOVs.

Using the Cranex 3D (Soredex, Tuusula, Finland) CBCT system, mandibular wax models were scanned with the following two FOVs:

FOV: 4×6 cm^2^; mA: 4, T:6.1s, kVp:90, voxel size: 0.136 mm^3^
FOV: 6×8 cm^2^; mA: 4, T:12.6s, kVp:90, voxel size: 0.2 mm^3^

Using the NewTom 3G (QR SRL Company, Verona, Italy) CBCT system, samples were scanned with the following FOVs:

FOV: 5×5 cm^2^; mA: 0.5, T:3.6s, kVp:110, voxel size: 0.16 mm^3^
FOV: 8×5 cm^2^; mA: 0.5, T:3.6s, kVp:110, voxel size: 0.25 mm^3^
FOV: 8×8 cm^2^; mA: 0.5, T:1.8s, kVp:110, voxel size: 0.33 mm^3^

Using 3D Promax (Planmeca OY, Helsinki, Finland) CBCT system, samples were scanned using the following FOV:

FOV: 8×8 cm^2^; mA:1.93, T:12 s, kVp:84, voxel size: 0.4 mm^3^

### 2.2 Evaluation of Images

**Fig. (1):**
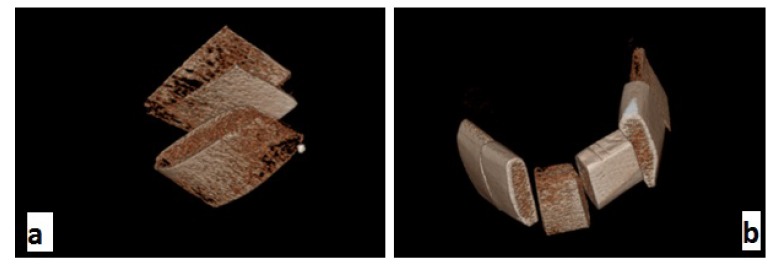
Three-dimensional reconstructed images of the second mandibular model taken by Cranex 3D ; a:in 4×6 cm^2^ FOV and b: in 6×8 cm^2^ FOV.

**Fig. (2):**
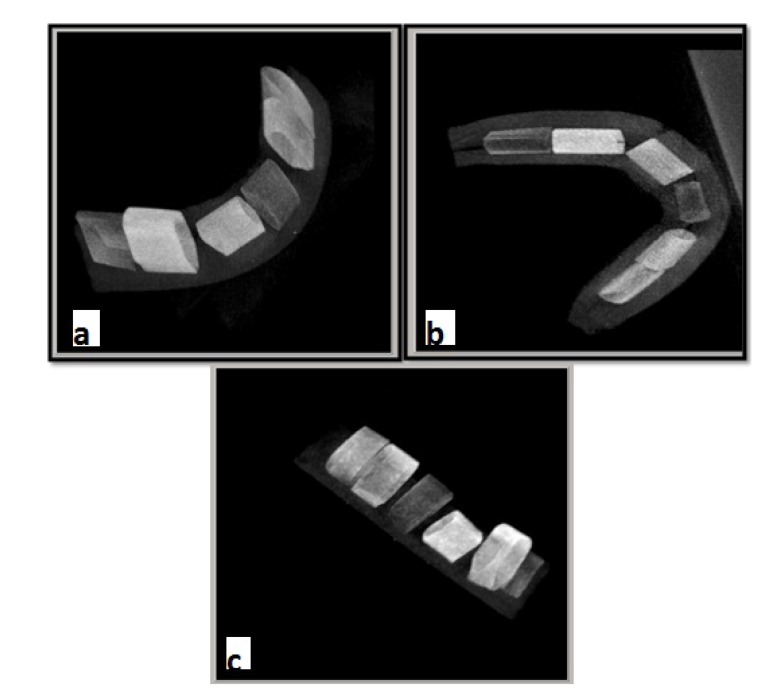
Three-dimensional reconstructed images of the second mandibular model taken by NewTom 3G; a: in 5×5 cm^2^ FOV and b: in 8×5 cm^2^ FOV and c: in 8×8 cm^2^ FOV.

**Fig. (3):**
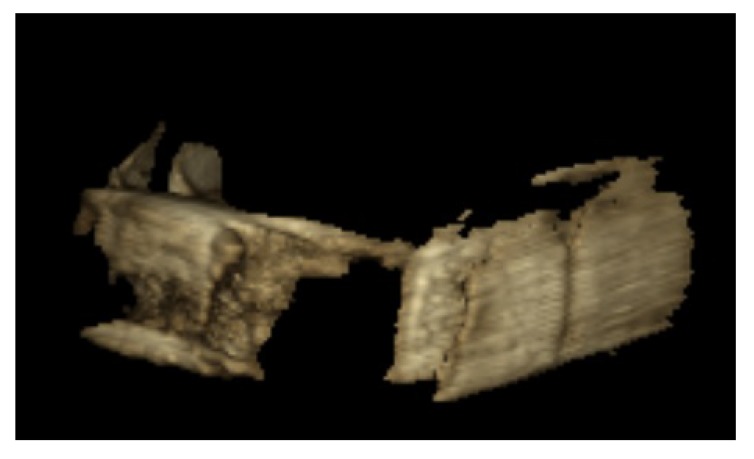
Three-dimensional reconstructed images of the second mandibular model taken by Planmeca CBCT system.

Using the respective software programs for each system, 3D images were reconstructed of all scans of each sample. The OnDemand 3D dental software (Soredex, Tuusula, Finland) was used in the Cranex 3D system (Fig. **1**). NNT Viewer software (Newtom, Verona, Italy) was used in NewTom 3G system (Fig. **2**) and Romexis software (Planmeca OY, Helsinki, Finland) was used in 3D Promax system for image analysis (Fig. **3**). All images were viewed in Multi-planar Reformation (MPR) format.

Two oral and maxillofacial radiologist viewed the images twice with a two-week interval regarding the observation of cortical bone on 3D scans.

### 2.3 Statistical Analysis

The data were analyzed using *SPSS* software (*SPSS* V.16, Chicago, IL, USA). Descriptive statistics were reported as frequency (percentage) and mean (standard deviation) values. In order to assess the intra- and inter-observer agreements, the kappa statistic was used.

The kappa statistic determines the possibility of one system to correctly predict the results according to uncertainty and chance. The kappa coefficient may range from −1 to +1. Cohen suggested that a score lower than 0.41 might be considered as a low agreement [10]. The kappa statistic was applied to assess the agreement in binary results of variables where the null hypothesis assumes no agreement between the results.

## 3 RESULTS

According to Table **1**, both inter- and intra-observer agreements were within the acceptable range.

Table **2** shows descriptive statistics of the cortical bone thicknesses in the three groups of less than 0.5 mm, between 0.6 mm and 1 mm and more than one millimeter. The descriptive statistics were reported as mean, standard deviation, minimum and maximum values of the cortical thickness.

Table **3** presents the data regarding the frequency and percentage of observing cortical bone with different thicknesses on CBCT scans taken with the three systems. For cortical bone thickness less than 1mm, the results of the three systems were compared using the kappa statistic.

As seen in Table 4, in samples with 0-0.5 mm cortical bone thickness, Cranex 3D system with 4×6 cm^2^ FOV showed higher efficacy for image reconstruction. The lowest efficacy belonged to 3D Promax system. Cranex 3D FOV: 4×6 cm^2^ is significantly in accordance with Cranex 3D 6×8 cm^2^ (kappa =0.8, *P* <0.001) and NewTom 3G Fov: 5×5 cm^2^ (kappa =0.4, *P* = 0.046). The binary results of Cranex 3D FOV: 6×8 cm^2^ were the same way as for NewTom 3G FOV: 5×5 cm^2^ (kappa =0.438, *P* = 0.032) and NewTom 3G FOV: 8×5 cm^2^ (kappa =0.341, *P* = 0.026). NewTom 3G FOV: 8×5 cm^2^ was also resulted similar to NewTom 3G FOV: 8×8 cm^2^ (kappa =0.654, *P* <0.001).

In samples with 0.6-1 mm cortical bone thickness, Cranex 3D with 6×8 cm^2^ FOV, Cranex 3D with 4×6 cm^2^ FOV, NewTom with 5×5 cm^2^ FOV and NewTom with 8×5 cm^2^ FOV showed higher efficacy (100%) for image reconstruction while 3D Promax followed by NewTom with 8×8 cm^2^ FOV showed the lowest efficacy, respectively. 3D Promax and NewTom 3G FOV: 8×8 cm^2^ were not in accordance statistically, the agreement coefficient was -0.05 (*P* = 0.758).

All systems evaluated in this study showed 100% efficacy for image reconstruction of cortical bone in thicknesses over one millimeter and were not significantly different in this respect [11].

## 4 DISCUSSION

Three-dimensional CBCT scans are increasingly used for treatment planning for an implant placement morphological assessment of the jaws and craniofacial structures and assessment of bone thickness and volume for orthognathic and reconstruction surgeries in the head and neck region due to trauma or pathologic lesions [12, 13].

However, the accuracy of reconstructed CBCT images has not been well investigated in previous studies [14].

Considering the importance of visualization of cortical bone on CBCT scans and since no previous study has evaluated the efficacy of CBCT systems for 3D image reconstruction of different thicknesses of cortical bone, this study aimed to assess the efficacy of three CBCT systems namely 3D Promax, Cranex 3D and NewTom 3G for 3D reconstruction of different thicknesses of cortical bone.

Studies on the accuracy of 3D image reconstruction by CT systems have shown that CT has limitations in image reconstruction of cortical bone in thicknesses less than one millimeter [11]. Thus, the current study assessed the efficacy of CBCT systems for image reconstruction of cortical bone in three different thicknesses of 0-0.5 mm, 0.6-1 mm and 1.1-1.5 mm. The results showed that CBCT systems were significantly efficient for 3D image reconstruction of cortical bone in thicknesses less than one millimeter, but the efficacy of the three systems was found to be different. The superiority of CBCT to CT for 3D reconstruction of cortical bone in thin thicknesses may be due to the fact that CBCT provides images with very small isotropic voxels, which result in higher spatial resolution of CBCT images [11, 15].

The three CBCT systems showed the greatest difference in the reconstruction of cortical bone with less than 0.5 mm thickness. Cranex 3D system with 4×6 cm^2^ FOV had the highest efficacy for 3D image reconstruction of cortical bone while 3D Promax system with 8×8 cm^2^ FOV had the lowest efficacy. 3D Promax had significantly lower efficacy than other systems. In the NewTom 3G system, 5×5 cm^2^ FOV had significantly higher efficacy than 8×5 cm^2^ and 8×8 cm^2^ FOVs for 3D image reconstruction of cortical bone. In the NewTom system, although 8×5 cm^2^ FOV was superior to 8×8 cm^2^ FOV in 3D image reconstruction of cortical bone, this difference was not statistically significant.

All three CBCT systems in the current study showed 100% efficacy for image reconstruction of cortical bone in thicknesses over one millimeter. In samples with 0.6-1 mm cortical bone thickness, Cranex 3D system with 4×6 cm^2^ FOV, Cranex 3D with 6×8 cm^2^ FOV and NewTom with 5×5 cm^2^ and 8×5 cm^2^ FOVs had significantly higher efficacy for cortical bone image reconstruction and all of them well visualized the cortical bone in 100% of the cases (cortical bone was visible on 100% of the reconstructed CBCT scans). In this group, 3D Promax system had the lowest efficacy followed by NewTom 3G with 8×8 cm^2^ FOV. The latter showed significantly higher efficacy in 3D image reconstruction of cortical bone than the 3D Promax system.

Ibrahim *et al*. [9] in their study on the effect of scanning parameters on CBCT trabecular bone microstructural measurements concluded that FOV was the most influential factor affecting the quality of final image, and the microstructure of trabecular bone was significantly more visible when smaller FOVs were used. They showed that when larger FOVs were used, trabecular thickness and trabecular spacing decreased, which complicated precise observation of this anatomical structure on the final image. Similarly, in our study, cortical bone (especially in samples with a thinner thickness of cortical bone) was better visualized on 3D CBCT scans when smaller FOVs were used. The difference between our study and that of Ibrahim *et al*, was that they used the mandible of a human cadaver and assessed the factors affecting visualization of microstructure of trabecular bone on cross-sectional images and reported their results based on observation of the number of trabeculae, thickness of trabeculae and the distance between them in different FOVs. However, we used bovine rib in order to obtain smaller thicknesses of cortical bone and compared the efficacy of three CBCT systems for visualization of different thicknesses of cortical bone.

In the clinical setting, the quality of reconstructed 3D CBCT scans and their capability in visualizing anatomical structures are affected by several factors, which are mainly related to the CBCT system used and the imaging parameters such as FOV, voxel size, exposure settings and some other technical issues; thus, different CBCT systems are expected to have different capabilities with regard to 3D reconstruction of an anatomical structure [16-18].

Comparison of different systems revealed that FOV significantly affects the quality of reconstructed images and must be taken into account when the goal is to assess anatomical structures on the reconstructed CBCT images [16]. Using a large FOV for imaging of dental arches and maxillofacial structures significantly decrease the spatial resolution and the quality of reconstructed 3D images [16]. A larger FOV results in less sharp reconstruction, which is attributed to the greater beam angulation in the superior and inferior volume areas and decreased contrast to noise ratio [16, 17].

Studies have mainly assessed the effects of FOV and voxel size on the quality of CBCT images. In smaller FOVs, voxel size is smaller as well. Although smaller voxel size increases the noise in the final image, due to its positive effect and providing images with higher spatial resolution, it increases the quality of the final image. Most studies on the effect of voxel size on the quality of final image have recommended using smaller voxel sizes.

Hedesin *et al*. [17] evaluated the efficacy of different CBCT systems and the effect of different FOVs on visualization of simulated periapical lesions on CBCT scans. They evaluated the mandible of slaughtered pigs using Accuitomo 3D, Scanora and New Tom 3G in 6, 9 and 12 mm FOVs. Based on their results, scans taken by Scanora system had the highest sensitivity for detection of periapical lesions while the other two systems were not significantly different. With regard to the size of FOV, although the diagnostic sensitivity for periapical lesions decreased in larger FOVs, this reduction was not significant; which is in contrast to our findings; however, it should be noted that they only evaluated cross-sectional images.

Wenzel *et al*. [19] and Melo *et al*. [20] assessed the parameters affecting the diagnostic accuracy of CBCT images in detection of root fractures and recommended the use of CBCT systems with smaller voxel size (smaller than 0.2 mm) and high resolution in cases suspected for root fracture.

Librizzi *et al*. [21] found that images with 0.2 mm voxel size had significantly higher accuracy for diagnosis of erosion of temporomandibular joint than those with 0.4 mm voxel size.

All previous studies evaluated the effect of voxel size on the quality of cross-sectional views of CBCT scans and only one study evaluated the reconstructed 3D images [9-11], none of the mentioned studies evaluated the effect of different parameters on visualization of cortical bone.

Maret *et al*. [22] evaluated the effect of voxel size on the accuracy of reconstructed 3D CBCT scans. They scanned extracted human teeth using Kodak 9500 3D system with 200 and 300 μm voxel sizes. Scans were also taken of the samples using micro-CT (Scanco Medical Xtreme CT) as the control group. Volumetric measurements in samples were made using AmIRA software. The results showed that under-estimation in tooth volumetric measurements made on reconstructed 3D CBCT scans was significantly higher when larger voxel sizes (300 μm) were used. A large voxel size of CBCT scans significantly under-estimated volumetric measurements compared to CBCT scans with small voxel size [22]. These results were similar to our article.

Measurement underestimation is described by the Partial Volume Effect (PVE) and is an important parameter affecting the spatial resolution and subsequently the quality of the final image [23, 24]. Also, 3D CBCT images include a wide range of gray value, which is affected by PVE [16]. When CBCT images are binarized, voxels are assigned either as bone or as marrow according to their gray values. Voxels with the data of both bone and marrow show a gray value between the two and thus, it is difficult to determine whether such a voxel displays bone or marrow. When larger voxel sizes are used, the voxel values under the influence of PVE can result in an image with thicker trabeculae or cause loss of thin trabeculae [25]. The CBCT systems with larger voxel size and higher spatial resolution are less affected by the PVE [24]. Increasing the voxel size decreases the sharpness of CBCT images, which reduces the diagnostic accuracy of anatomical structures [24].

The current study also showed that in CBCT systems with different FOVs, smaller FOVs and consequently smaller voxel sizes better visualized cortical bone, especially in very thin thicknesses. Cranex 3D system with 4×6 cm^2^ FOV with 0.136 mm^3^ voxel size had significantly greater ability for visualization of cortical bone with less than 0.5 mm thickness compared to 6×8 cm^2^ 2 FOV with 0.2 mm^3^ voxel size. Three different voxel sizes of the NewTom 3G system were evaluated and it was found that 5×5 cm^2^ FOV with 0.16 mm^3^ voxel size had significantly higher efficacy for 3D image reconstruction of cortical bone with less than one millimeter thickness compared to 8×5 cm^2^ FOV with 0.25 mm^3^ voxel size and 8×8 cm^2^ FOV with 0.33 mm^3^ voxel size.

## CONCLUSION

Most CBCT systems have high efficacy for 3D image reconstruction of cortical bone with thicknesses over 1 mm while they have poor efficacy for image reconstruction of cortical bone with less than 0.5 mm thickness. Thus, for accurate visualization of anatomical structures on CBCT scans, systems with smaller FOVs and consequently smaller voxel size are preferred.

## Figures and Tables

**Table 1:** Results of Kappa statistic (*P*-value) for assessing the intra and inter observer agreements.

System	Intra-Observer Agreement for the First Observer	Intra-Observer Agreement for the Second Observer	Inter-Observer Agreement
Soredex FOV: 4×6cm2	1.000 (<0.001)	1.000 (<0.001)	1.000 (<0.001)
Soredex FOV: 6×8cm2	0.926 (<0.001)	0.920 (<0.001)	1.000 (<0.001)
Planmeca FOV: 8×8cm2	0.970 (<0.001)	0.968 (<0.001)	0.969 (<0.001)
NewTom FOV: 5×5cm2	0.800 (<0.001)	1.000 (<0.001)	0.804 (<0.001)
NewTom FOV: 8×5cm2	0.961 (<0.001)	0.959 (<0.001)	0.963 (<0.001)
NewTom FOV: 8×8cm2	1.000 (<0.001)	0.898 (<0.001)	0.866(<0.001)

**Table 2:** Descriptive statistics for cortical thickness in its different categories.

Cortical Thickness(mm)	N	Mean (mm)	Std. Deviation	Minimum (mm)	Maximum (mm)
0-0.5	24	.400000	.0884652	.2000	.5000
0.6-1	24	.729167	.1122078	.6000	.9000
1.1-1.5	24	1.295833	.1781039	1.1000	1.5000
Total	72	.808333	.3945937	.2000	1.5000

**Table 3:** Frequency (percentage) of visibility of cortical bone.

System	≤ 0.5 mm	0.6-1 mm	>1 mm
Soredex FOV: 4×6cm^2^	18 (75%)	24 (100%)	24 (100%)
Soredex FOV: 6×8cm^2^	16 (66.7%)	24 (100%)	24 (100%)
Planmeca FOV: 8×8cm^2^	1 (4.2%)	22 (91.7%)	24 (100%)
NewTom FOV: 5×5cm^2^	16 (66.7%)	24 (100%)	24 (100%)
NewTom FOV: 8×5cm^2^	7 (29.2%)	24 (100%)	24 (100%)
NewTom FOV: 8×8cm^2^	4 (16.7%)	23 (95.8%)	24 (100%)

**Table 4:** Comparison and level of agreement between different CBCT systems for 3D visualization of cortical bone with thicknesses 0.5 mm or less.

System 1	System 2	≤ 0.5 mm	
		Kappa	*P*-Value
Soredex FOV:4×6 cm^2^	Soredex FOV: 6×8cm^2^	0.800	<0.001
	Planmeca FOV: 8×8cm^2^	0.029	0.555
	NewTom FOV:5×5cm^2^	0.400	0.046
	NewTom FOV:8×5cm^2^	0.241	0.070
	NewTom FOV:8×8cm^2^	0.125	0.206
Soredex FOV: 6×8 cm^2^	Planmeca FOV: 8×8cm^2^	0.043	0.47
	NewTom FOV:5×5cm^2^	0.438	0.032
	NewTom FOV:8×5cm^2^	0.341	0.026
	NewTom FOV:8×8cm^2^	0.182	0.121
Planmeca FOV: 8×8 cm^2^	NewTom FOV:5×5cm^2^	0.043	0.47
	NewTom FOV:8×5cm^2^	-0.079	0.512
	NewTom FOV:8×8cm^2^	-0.071	0.648
NewTom FOV:5×5 cm^2^	NewTom FOV:8×5cm^2^	0.341	0.026
	NewTom FOV:8×8cm^2^	0.182	0.121
NewTom FOV:8×5 cm^2^	NewTom FOV:11×13cm^2^	0.654	0.001
